# Immersive virtual reality for upper limb rehabilitation: comparing hand and controller interaction

**DOI:** 10.1007/s10055-022-00722-7

**Published:** 2022-12-02

**Authors:** M.-Carmen Juan, Julen Elexpuru, Paulo Dias, Beatriz Sousa Santos, Paula Amorim

**Affiliations:** 1 Instituto Universitario de Automática e Informática Industrial, Universitat Politècnica de València, C/Camino de Vera, S/N, 46022 Valencia, Spain; 2 Department of Electronics Telecommunications and Informatics (DETI), University of Aveiro, Aveiro, Portugal; 3 Centre Region Rehabilitation Medicine Centre Rovisco Pais (CMRRC-RP), Tocha, Portugal

**Keywords:** Serious games, Virtual reality, Hand gestures, Hand tracking, Standalone headsets, Motor rehabilitation

## Abstract

Virtual reality shows great potential as an alternative to traditional therapies for motor rehabilitation given its ability to immerse the user in engaging scenarios that abstract them from medical facilities and tedious rehabilitation exercises. This paper presents a virtual reality application that includes three serious games and that was developed for motor rehabilitation. It uses a standalone headset and the user's hands without the need for any controller for interaction. Interacting with an immersive virtual reality environment using only natural hand gestures involves an interaction that is similar to that of real life, which would be especially desirable for patients with motor problems. A study involving 28 participants (4 with motor problems) was carried out to compare two types of interaction (hands vs. controllers). All of the participants completed the exercises. No significant differences were found in the number of attempts necessary to complete the games using the two types of interaction. The group that used controllers required less time to complete the exercise. The performance outcomes were independent of the gender and age of the participants. The subjective assessment of the participants with motor problems was not significantly different from the rest of the participants. With regard to the interaction type, the participants mostly preferred the interaction using their hands (78.5%). All four participants with motor problems preferred the hand interaction. These results suggest that the interaction with the user’s hands together with standalone headsets could improve motivation, be well accepted by motor rehabilitation patients, and help to complete exercise therapy at home.

## Introduction

More than 17 million people suffer a stroke each year (Krishnamurthi et al. 2013). Due to advances in medicine, stroke mortality has been decreasing, resulting in an increasing number of survivors with motor, psychological, cognitive, social, and economic handicaps that have a negative impact on their quality of life (Lawrence et al. 2001). Six months after a stroke, a large percentage of survivors have motor deficits including hemiparesis (50%) and dependence in activities of daily living (26%) (Go et al. 2013).

At an early stage after stroke, survivors usually have access to rehabilitative care in hospitals, clinics, rehabilitation centers, and other facilities. After those first months, since most patients are medically discharged and do not have the possibility of maintaining treatments, they are encouraged by doctors and therapists to practice exercises at home. However, adherence to exercises that are performed at the patient's home is usually low, due to lack of motivation, low tolerance for effort, fatigue, or musculoskeletal changes such as joint stiffness or spasticity (Jurkiewicz et al. 2011).

Virtual Reality (VR) with its potential for creating fun and immersive environments and games has emerged as a promising path to increase motivation and encourage survivors to practice motor rehabilitation (Dias et al. 2019; Jonsdottir et al. 2021). This path can replace boring mandatory exercises with entertaining games or activities that are highly customizable to the patient’s own hobbies and tastes. Besides increasing motivation, the use of VR with tracking technologies to monitor gestures will enable the quantification of movements. The use of additional measures for evaluating the general quality of life of patients will, in turn, provide health professionals with the possibility of monitoring the patients’ recovery.

VR has already been successfully used to help patients bear pain and withstand other disease treatments (Schneider and Hood 2007; Patterson et al. 2010; Maani et al. 2011; Baños et al. 2013) as well as to recover from stroke (Cho et al. 2014; Covarrubias et al. 2015). VR offers great potential for rehabilitation (Liu et al. 2016; Laver et al. 2017) since it motivates the patients, allows immersion in engaging virtual environments while providing multiple stimuli, and promotes the improvement of cognitive and motor capacities. Affordable sensors for gesture tracking have been studied and developed (mainly in the gaming industry), which can be explored for rehabilitation (Piron et al. 2009; Covarrubias et al. 2015). This synergy between affordable technology and the benefits it offers makes virtual reality systems tools with great potential for the rehabilitation of stroke, one of the leading causes of disability worldwide.

Telerehabilitation is a promising tool for minimizing the discontinuity of treatment after hospital discharge and for empowering patients to manage their health via interaction with remote rehabilitation professionals (Amorim et al. 2020). VR systems fulfill the fundamental principles of rehabilitation: environments with diversity in stimuli, task-oriented training, intensity, biofeedback, and motivation. All of these are fundamental factors for the success of rehabilitation therapy (Dias et al. 2019). The following benefits of using VR in rehabilitation have already been identified (Laver et al. 2017): increased motivation and collaboration of patients during rehabilitation programs, better performance, neuroplasticity stimulation, improvement of cognitive functions and of the affected limb, and greater autonomy in activities of daily life. Moreover, when combining virtual reality and traditional rehabilitation, stroke patients showed significantly greater improvement in their activities of daily life than those patients treated only with traditional rehabilitation therapy (Kim 2018). This makes VR an interesting tool for therapy. For example, VR therapies have demonstrated to be effective in pain management, in both sick and healthy subjects and have also shown to have very few side effects compared to other more aggressive therapies (Liu et al. 2016). Therefore, VR serious games could be used as a tool to train stroke survivors to monitor their health under the supervision and control of doctors.

The main objective of the work presented here is to develop and test a VR application for upper limb rehabilitation with hand interaction and visualization using a standalone headset in order to identify its strengths and limitations. Three different games were developed mapping simple gestures that are included in Enjalbert’s test (Enjalbert et al. 1988), which is a well-known scale that is used for the functional assessment of the upper limb mobility. We carried out a study to test the developed games regarding performance outcomes and subjective perception with 24 healthy people and 4 people with motor problems. The hypotheses to be corroborated in our study were the following: H1: Users will rate the games positively; H2: There will be no significant differences in the performance of the participants when using controllers or hands; H3: There will be no significant differences in the performance of the participants during the study based on their gender; H4: Participants will express their preference for the use of their hands for interaction. The remainder of this paper is organized as follows: we describe the application and the games developed. For the study, we present and discuss the main results, and finally we draw conclusions and present our ideas for future work.

## Related work

Virtual reality for motor rehabilitation has received a fair amount of attention from the scientific community, as the latest published review articles demonstrate (Levac et al. 2019; Kim et al. 2020; Høeg et al. 2021; Amirthalingam et al. 2021).

For motor rehabilitation of upper limbs using VR, the patients’ movement must be tracked so that this information can be used by the VR system and the patients can receive feedback on their actions. For motion tracking, the two most widely used technologies are based on wearable and vision sensors. Wearable sensors can be classified into those that use an exoskeleton and those that use data gloves.

The feedback that reaches the user can be visual, auditory, haptic, or a combination of these. Haptic feedback can be classified into tactile or force feedback. Tactile feedback comes to the users through the sense of touch and lets them know if a surface is rough or smooth, or hot or cold. The force feedback allows the users to have the sensation of grabbing or dropping objects.

The first studies on the use of VR for the rehabilitation of motor problems date back to the late 90s and early 2000s (Prisco et al. 1998; Boian et al. 2003; Sveistrup 2004; Holden 2005; Rose et al. 2005). In one of the first works (Prisco et al. 1998), visual, auditory, and haptic feedbacks were already included. For visual feedback, they used a HMD. Auditory feedback was achieved using headphones. For haptic feedback, an arm and hand exoskeleton that was developed in PERCRO laboratory was used. In 1998, the contribution of Prisco et al. (1998) was to show the potential of VR and haptic feedback for teaching motor skills.

Different systems that use wearable sensors have been presented (Dimbwadyo-Terrer et al. 2016; Calabrò et al. 2019). Dimbwadyo-Terrer et al. (2016) used VR and a CyberTouch™ data glove, for therapy after spinal cord injury. Nine patients participated in their study, which compared the VR system and the data glove versus traditional rehabilitation over two weeks. No statistically significance was found between the two groups. However, the authors argued that the data glove group seemed to offer some clinical changes. Calabrò et al. (2019) compared the neurophysiological and clinical effects of Amadeo™ (https://tyromotion.com/en/products/amadeo) for hand therapy versus intensive occupational therapy. Amadeo™ is a robotic and sensor-based rehabilitation device for use with either hand. Calabrò et al. concluded that there were neurophysiological evidences of the therapeutic impact of the treatment using Amadeo™ in the recovery of hand function in patients with chronic stroke.

As our proposal is based on hand tracking using vision sensors, special attention has been paid to this technology. The release of the Microsoft^®^ Kinect™ sensor in November 2010 represented a turning point in motion tracking and since that moment numerous systems have been developed with diverse applicability. One of the fields to which Microsoft^®^ Kinect™ has been applied is motor rehabilitation for the upper limbs (Wen et al. 2014; Cho et al. 2016; Ballester et al. 2017; Aşkın et al. 2018; Oña et al. 2018; Reggente et al. 2020). Wen et al. (2014) used the SIGVerse virtual platform Microsoft^®^ Kinect™ for motion tracking, and a HMD (Vuzix) for visual feedback. They also captured EMG (electromyography) signals. An elbow flexion study was conducted to test the performance and feasibility of their system. Wen et al. (2014) concluded that the system had excellent feasibility, but there were some aspects that needed improvement. Cho et al. (2016) used Microsoft^®^ Kinect™ for grasping assessments and used the box and block test (Mathiowetz et al. 1985), which is an assessment tool to estimate the ability of patients to grasp and carry objects. Cho et al. (2016) used a screen monitor for visual feedback. The Microsoft^®^ Kinect™ was placed approximately 1 m above a table. A study involving nine patients was carried out to compare their VR system and the real box and block test. Cho et al. (2016) concluded that all of the patients were able to move some blocks in both conditions, but the number of blocks moved was significantly lower in the virtual condition. As Cho et al. (2016) argued, the recognition of gestures was limited with Microsoft^®^ Kinect™, a fact that could significantly affect the results. Ballester et al. (2017) carried out a study to compare the effectiveness of home therapy using VR therapy versus occupational therapy to induce motor recovery of the upper limbs. A screen monitor was used for visual feedback. Microsoft^®^ Kinect™ was used for motion tracking, and a pair of data gloves with built-in bend sensors was in charge of capturing the flexion of the fingers. A total of thirty-five patients with chronic stroke participated, undergoing three weeks of home treatment. Ballester et al. (2017) concluded that, in the chronic stages, motor rehabilitation using VR facilitates functional improvements, accompanied by neuroplastic changes. Aşkın et al. (2018) carried out a study comparing two groups of patients. In Group A, the patients received 20 physical therapy sessions plus 20 Kinect VR training sessions. Group B received only 20 sessions of physical therapy. Aşkın et al. (2018) concluded that the joint use of physical therapy and VR training can contribute to the improvement of upper limb motor function and active range of motion in chronic stroke patients.

Another revolutionary device was Leap Motion (released in 2013), which allows tracking of the fingers of the hand more precisely than Microsoft^®^ Kinect™. This device has also been used for the development of numerous applications applied to different fields. One of the fields to which Leap Motion has been applied is motor rehabilitation for the upper limbs (Wang et al. 2017; Colombo et al. 2019; Dias et al. 2019). Dias et al. (2019) used the Oculus Rift DK2 for visual feedback and the Leap Motion sensor for hand tracking. The audio feedback was achieved using a speaker that was placed in front of the patient. Twelve patients in several phases of recovery and suffering from different stroke sequelae participated in their study for the comparison of immersive and non-immersive versions of the system. Dias et al. (2019) concluded that their system was very well received by therapists, doctors, and patients, and its main benefit was the increase in patients’ motivation for recovery by using relaxed and fun environments. Takeo et al. (2021) also presented a VR system for motor learning using a HMD (Oculus Rift) and a hand tracking system (Leap Motion). Wang et al. (2017) presented a VR system for motor recovery of upper limbs after a subacute stroke. They use the screen of a personal computer for the visual feedback and the Leap Motion for hand tracking. They compared VR training along with conventional occupational rehabilitation (*N* = 26) versus only conventional rehabilitation during four weeks. The VR training group had significantly better improvement than the control group. Colombo et al. (2019) used Leap Motion for hand tracking and added musical sonification for the rehabilitation of hand motor function. The participants sat in a wheelchair with a transparent side-table on the side to be trained. The forearm and elbow could rest on the side-table during training. A computer equipped with an external loudspeaker was used for audio feedback. The Leap Motion was located about 25 cm below the level of the side-table. The participants did not receive visual feedback on the computer screen. A study involving 15 stroke patients and 15 healthy individuals was carried out, proving that their system was feasible.

Our proposal is different in terms of the hardware used in previous works (Wang et al. 2017; Colombo et al. 2019; Dias et al. 2019; Takeo et al. 2021). For visual feedback, they used Oculus Rift that has to be connected to a computer (Dias et al. 2019; Takeo et al. 2021) or a computer screen (Wang et al. 2017). We used the Oculus Quest, which is a standalone headset. All of these works use Leap Motion sensor for hand tracking, which is an external device that has to be connected to a computer (Wang et al. 2017; Colombo et al. 2019; Dias et al. 2019; Takeo et al. 2021). In our case, we used the hand tracking capabilities incorporated in Oculus Quest. Previous works focus mainly on using their systems for therapy and comparing them with another type of therapy (Wang et al. 2017; Colombo et al. 2019). Few works focus on comparing different VR systems or differences in their visualization or interaction. One of these works was presented by Dias et al. (2019), in which immersive and non-immersive versions were compared. Our study compares hand versus controller interaction. Therefore, to our knowledge, our proposal represents another turning point in the use of technologies for motor rehabilitation. It is the first system to propose the use of a standalone headset and interaction using the user's own hands (without the need to wear anything on them) for upper limb rehabilitation.

## Design and development of the VR application

### Design

The application consists of three games. The Enjalbert test (Enjalbert et al. 1988) was used as the basis for its design. The Enjalbert test consists of five main exercises:

- Raise the affected arm to a specific height and hold it in a static position
- Bring the affected hand to the mouth
- Open and close the affected hand
- Touch the index finger and middle finger with the thumb
- Touch the ring finger and little finger with the thumb

All of these exercises are designed to test upper body mobility along with hand mobility and grip strength. The first three types of exercises were incorporated in our application.

### Description of the application and its games

When the application starts, a main menu is displayed that allows the user/patient to select which hand to train and which game to play. During the exercises, if a hand is used that is different from the one selected, a warning message appears indicating that the wrong hand is being used and that the user cannot advance in the exercise. The exercise data is automatically stored during the game.

**Fig. 1:**
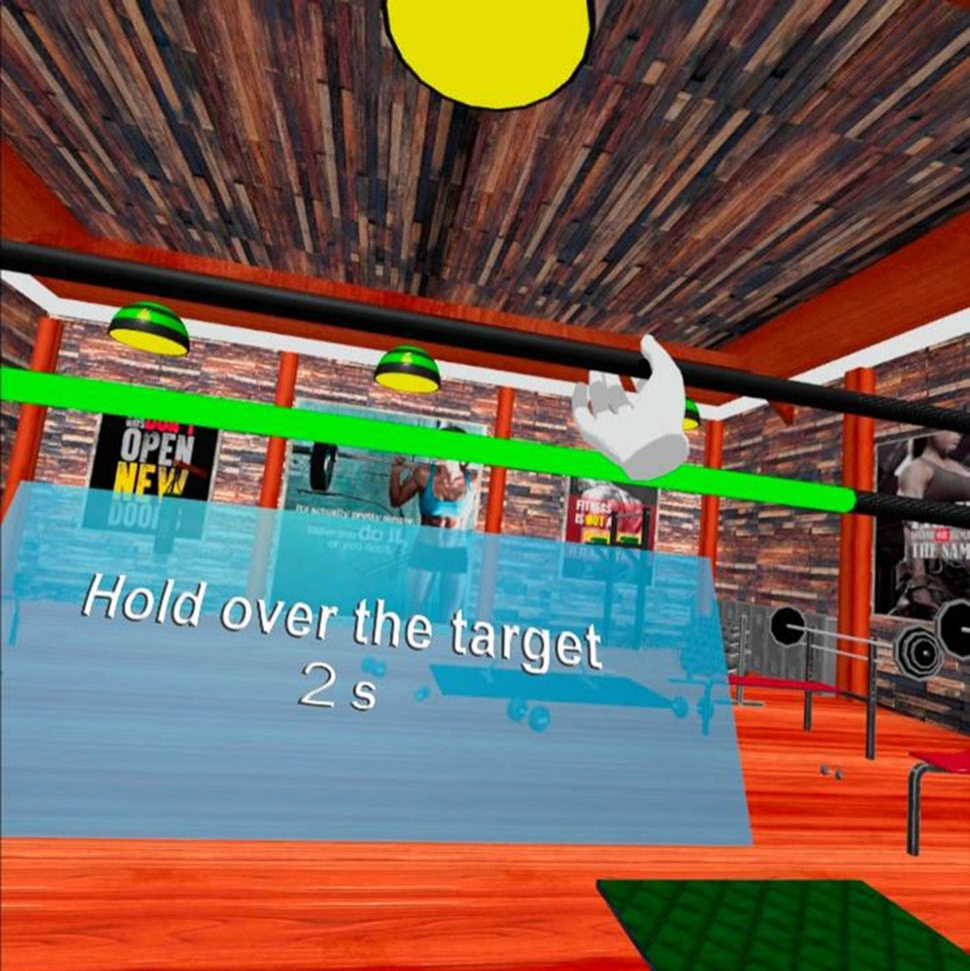
Example of the lifting exercise

The *first game* consists of raising the affected hand above a target height and keeping it in that position for a specified time. After successfully performing the exercise, the user should lower a barbell below that height and rest that hand for a few seconds. The default settings for the lifting height, times to hold, times to rest, and iterations are 60 cm, 3 s, 3 s, and 3 times, respectively. These settings and those of the other games can be personalized at will by the specialist depending on the patient’s needs. To make the exercise more attractive to the user, the virtual environment simulates a gym in which the user must lift a barbell above a target bar (which changes from red to green when the barbell is lifted above it). In addition to the height indicator shown as the target bar, the instructions that the user must follow also appear. These include the duration in seconds for holding the barbell above the target bar or resting after reaching it. The game ends after a specified number of repetitions (Fig. 1). Lifting the barbell is achieved using the user’s hand or the controllers. In either case, a virtual hand appears on the screen. The users have to perform a movement that is similar to what they would make if they were using a real barbell (grasping the barbell from below and pushing it upwards). The hand can be placed in any position as long as it makes contact with the bar. This position has not been limited to a central area of the bar, nor is the user required to open the affected hand as some patients might not be able to close it.

**Fig. 2:**
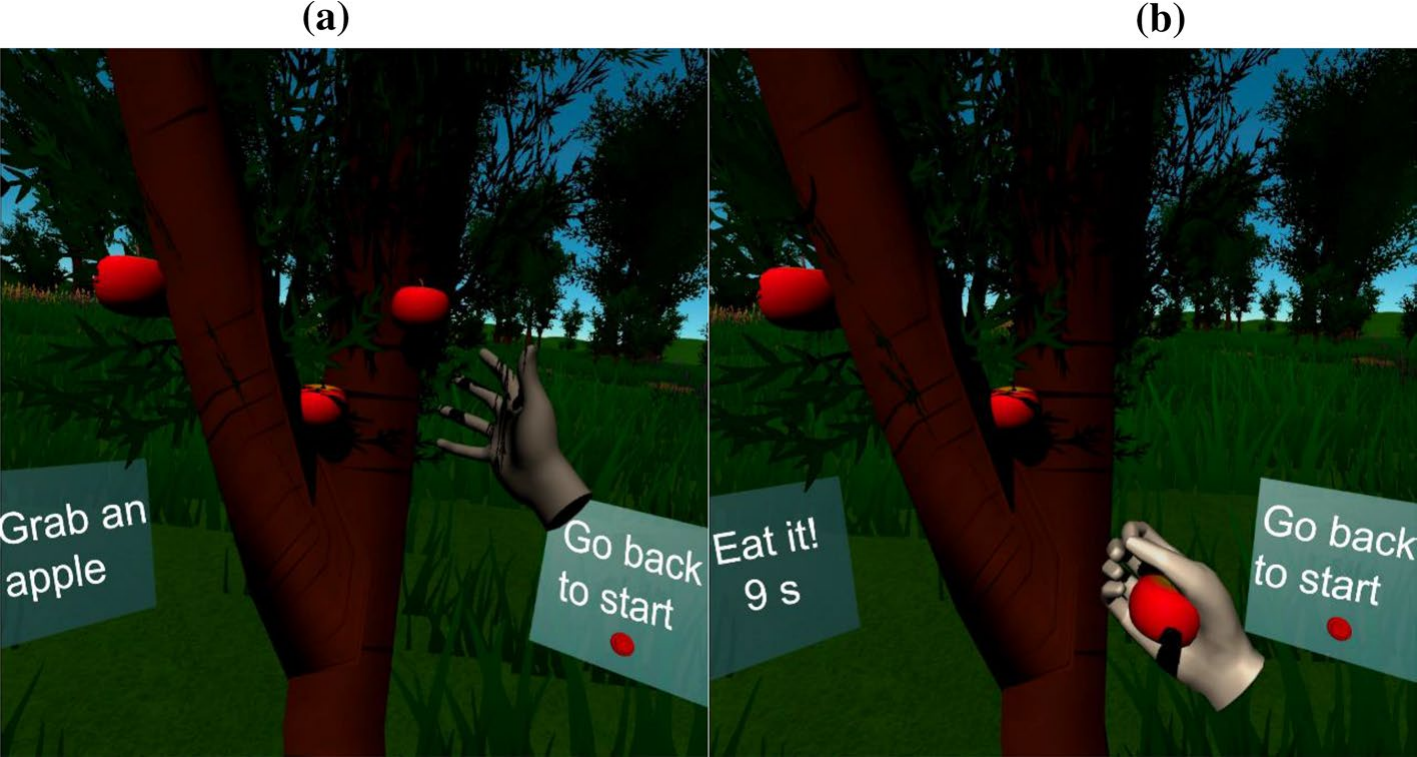
Two examples of the “eating an apple” exercise: **a** using hands and **b** using controllers

The *second exercise* consists of stretching and bending movements of the elbow in order to bring the hand to the mouth. After doing an iteration of this movement, the user must rest. In this exercise, the user is also placed in a context that simulates a suitable and attractive scenario for the exercise. Specifically, a field with an apple tree with red apples was modeled. The user stands in front of the tree and is instructed on how to perform the exercise (Fig. 2). The user must stretch to reach one of the apples and then bring it to his/her mouth. As in the previous exercise, the user is not required to perform the grabbing gesture (closing the hand). Therefore, touching any apple is enough to pick it up. As soon as an apple is reached, the user has a predefined time (which is configurable) to bring it to his/her mouth. In Fig. 2b, the number 9 indicates the time left in seconds to complete the action of eating the apple successfully after having grabbed it. If the user is not successful, the apple falls from the user’s hand and returns to its original position. Although users can tilt or move their head freely using our application, they were informed that the correct movement is to keep their head steady and move only their arms (as tilting the head would require less arm lift).

**Fig. 3:**
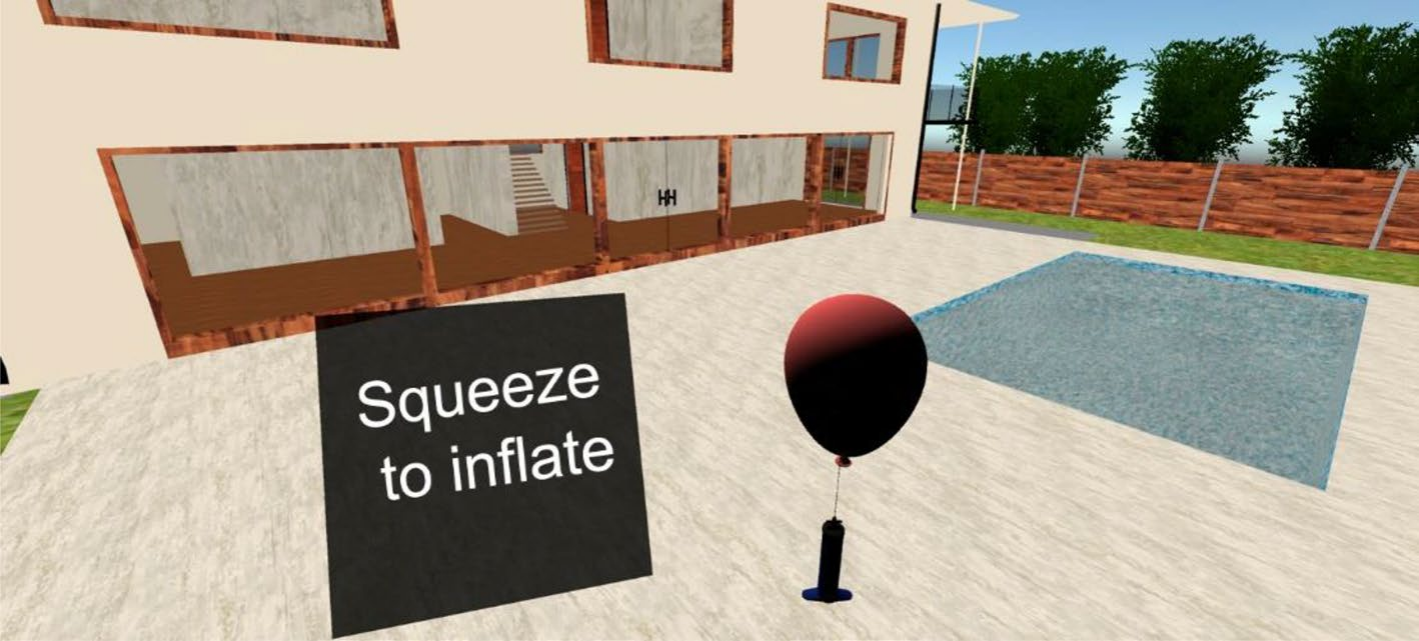
Example of the exercise for opening and closing the affected hand

The *third exercise* consists of evaluating the user’s ability to close the affected hand by asking him/her to close it tightly. For this exercise, the chosen environment was the exterior of a house with a pool in which the user prepares a party and must inflate a balloon with a hand-held inflator (Fig. 3). To do this, the user must hold the inflator while squeezing the ball to inflate the balloon. At this point, the screen prompts the user to open his/her hand. By doing so, the user completes the first iteration. Then, the rest time begins before moving on to the next iteration. The exercise is completed by performing the established number of iterations. Additional instructions are provided: "You must imagine that you are really grasping a ball and must close your fingers like a claw, that is, stretch and flex the phalanges until the tips of all of your fingers practically touch." In general, the users had no problem performing this exercise with this instruction. In the interactions of the two previous exercises, there were no differences when using hands or controllers. However, in the third exercise, the interaction was different. To select the buttons on the controllers to be pressed, we analyzed what a real use of an inflator would be like. The most natural gesture when using an inflator like the one simulated in our exercise is to close the hand by flexing the index, middle, ring, and little fingers. We analyzed the available buttons of the controllers to simulate a controller as an inflator. It was observed that the hand trigger is the button that best represented this action. For this reason, when using controllers, the hand is considered to be tightly closed when the hand trigger of the controller is properly pushed. The finger that has pressed the trigger is not checked, but the most natural and intuitive way is for all of the fingers to be flexed and for the finger that presses the trigger to be the middle finger (for users familiar with Oculus controllers) or the index finger (if the index trigger is not used).Using more buttons simultaneously did not provide any more similarity to the real action and could mislead users who are less familiar with the use of controllers of this type. When using hand tracking, the Oculus SDK functionality was used for this purpose, which enables hand rigging and associated functions. In our implementation, we use a function (GetFingerPinchStrength) for each finger, and, when the value of every single one of them is greater than a specified threshold (personalized at will), the hand is considered closed in terms of the exercise.

### Hardware and software

The application was developed and tested on Oculus Quest (https://www.oculus.com), which is a standalone device that does not need to be connected to a computer or mobile phone. The operating system is Android. It also has two wireless controllers for an intuitive interaction, but, for our work, the most remarkable feature is its hand tracking capabilities.

The application was developed using the Unity game engine (https://unity.com). Several elements of the Unity Asset Store were also used as a base. These were adapted or modified to adjust each of the desired environments. Some of the models were modified or modeled using Blender. For correct installation, a series of dependencies that have to do with both the operating system and the Unity engine must be resolved in order to work with Oculus Quest. For the operating system, we highly recommend having Android Debug Bridge installed since it allows full control of the device via the command console. This may seem trivial when the programmer has direct access to files through the system explorer, but using this method is going to be the only possible way to get debug logs while running the application in Oculus Quest. For the Unity editor, the Android Build Support module must be added to be able to build applications for this operating system. In the Unity editor, Package Manager window should be selected. In this window, then XR Plugin Management should be selected, and then Oculus should be selected in Plugin Providers. The AndroidManifest must be accessible since any necessary permission modifications must be specified in this XML file (e.g., to give permissions to track hands or write files). The developer mode must be enabled on the Oculus device. To do this, the programmer must register on the developer’s website. This registration allows the installation of applications of unknown origin on the device. The Oculus SDK installation can be done from the Unity Asset Store (https://assetstore.unity.com). For ease of use, the open source program SideQuest (https://sidequestvr.com) was used to allow a more direct management of the files in the internal storage of Oculus Quest.

## Study

The hypotheses to be corroborated in our study were introduced in the Introduction section and are the following: H1: Users will rate the games positively; H2: There will be no significant differences in the performance of the participants when using controllers or hands; H3: There will be no significant differences in the performance of the participants during the study based on their gender; H4: Participants will express their preference for the use of their hands for interaction.

### Measures

#### Performance with the two types of interaction

The application stores the Number of Attempts in the three games as well as the Total Time used. These are the dependent variables that were used as performance variables.

#### Subjective measures

After playing the first time, the participants fill out a questionnaire consisting of twenty-eight questions. Twenty-two of the questions were subjective and related to the use of the application; four questions were related to their familiarity with gaming devices, virtual reality applications, and headsets; and two questions were open-ended. The twenty-two subjective questions were on a 7-point Likert-type scale, ranging from 1 “Totally disagree” to 7 “Totally agree”. All of the items were formulated in a positive manner. The questionnaire was specifically designed for this study and was based on previously used questionnaires (Brooke 1996; Rodriguez-Andres et al. 2018; Munoz-Montoya et al. 2019). The second questionnaire was designed to assess the users' preference for the type of interaction. It consists of the two following questions: What type of interaction do you prefer (controllers or hands)? Why?

### Experimental conditions and design

The objective of the study was to compare the performance in the games when using the two different types of interaction (controllers vs. hands), as well as the subjective perception of the participants. Therefore, there are two conditions:

- Controllers: The participants used the controllers.
- Hands: The participants used their hands.

To compare these two conditions, the sample was divided into two groups (within-subjects design with half of the participants starting with one of the two conditions in order to avoid bias):

- Group A (controllers): The participants used the controllers for their first use and their hands for the second use. The second use was only performed to know the opinion of the participants regarding the two types of interaction.
- Group B (hands): The participants used their hands for their first use and the controllers for their second use. The second use was only performed to know the opinion of the participants regarding the two types of interaction.

### Procedure

The study was conducted during the COVID-19 pandemic. The sessions were carried out following a sanitary protocol prior to the use of the material by the users. The protocol followed throughout the session with each participant was as follows:

- Before starting the session, the workspace was defined in Oculus and a chair was placed in the center of the room. It was confirmed that there was enough space to guarantee mobility, especially on the right side where the exit button is always placed.
- All equipment (headset and controllers) were cleaned with antibacterial wipes before and after each use.
- The subjects were given a brief introduction to the exercises, including the objectives that they would have to meet in each game and the reason for choosing that game for those movements.
- The subjects were provided with hydroalcoholic gel to wash their hands.
- The subjects were provided with vinyl gloves for both hands.
- They were asked if they were right-handed or left-handed and if they had any kind of pathology affecting their hands.
- The controllers were shown to them. The correct way to grasp them and which buttons they were going to use throughout the different moments of the exercises were explained.
- Before putting on the headset, a VR Face Mask was put on to avoid contact of the headset with the skin as much as possible. It was confirmed that the subjects' vision was not obstructed or limited by the mask.
- The headset was adjusted explicitly indicating that it must squeeze, but not hurt. Before finally adjusting the headset, the participants had to confirm that they were reading and seeing clearly. The distance between their eyes in the headset was adjusted when necessary.
- The subjects were told how to get from the main menu to the application. This was done to familiarize them with the controllers and the environment before using the application.
- The subjects were instructed to carry out the three exercises in the same order (from first to third), first with one method of interaction and later with the other, depending on the participation group to which they belonged. In all cases, the subjects used their dominant hand for the exercises, except in those cases in which a pathology was associated with a specific hand. The comments, complaints, or suggestions made by the subjects during the tests were also registered by the supervisor.
- At the end of the session, the subjects themselves discarded both the VR Face Mask and the gloves in order to avoid any kind of contact.
- The subjects were instructed to fill out the online questionnaires, one for each type of interaction. The supervisor was present to clarify any doubts that the questions could generate, but without influencing the answers. After playing for the first time using one of the interaction types, the participants filled out an online questionnaire about their subjective perception. After using the two types of interaction, the participants were asked about their preference and why they preferred one type of interaction over the other.
- At the end of the session, all of the material was cleaned once more with wipes and hydroalcoholic gel before moving on to the next subject.

The material used for hygiene measures was as follows:

- Antibacterial wipes for medical use.
- Hygienic vinyl gloves.
- Hygienic breathable VR face mask for Oculus Quest to avoid sweat and contact with the headset.
- Hydroalcoholic disinfectant gel.

### Participants

The sample involved 28 subjects. This sample consisted of 15 men (53.6%) and 13 women (46.7%), ranging in age between 8 and 73 years old with a mean (standard deviation) of 40.61 (19.47).

**Fig. 4:**
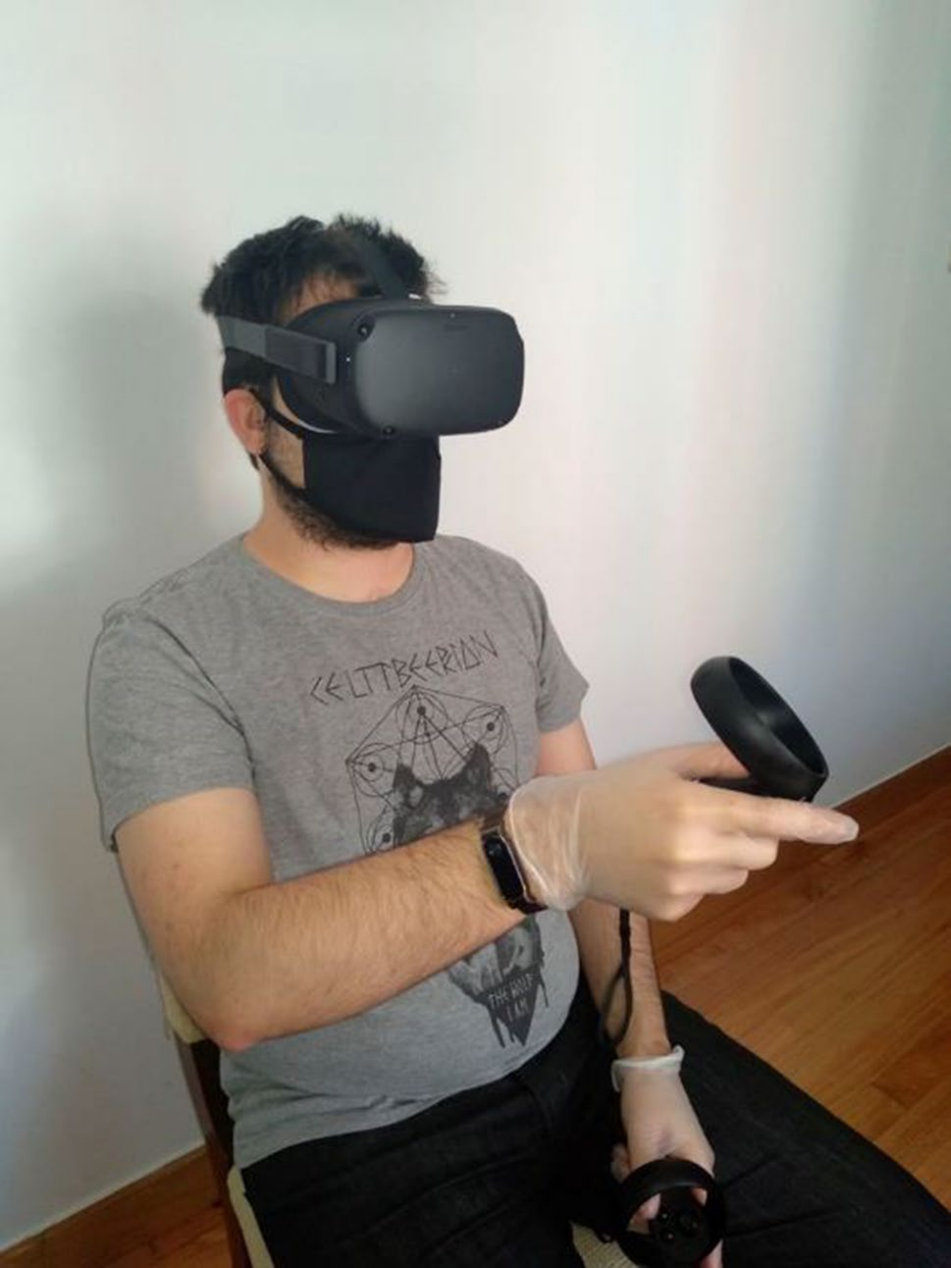
Example of a participant using the controllers

The 28 participants were distributed in a balanced way so that Group A consisted of 14 users (50%) and Group B consisted of the same number, 14 (50%). Among the members of both groups there were also subjects who suffered from some type of pathology that could hamper the task to be performed. Specifically, there was a 46-year-old man with Parkinson's disease, a 71-year-old man with osteoarthritis, a 59-year-old woman with essential tremors, and a 73-year-old woman with tendinitis. These participants with motor problems were included with the rest of the sample to determine whether or not they were outliers. This aspect is further discussed in the results and discussion sections. Figure 4 shows a participant.

The participants or their parents were informed about our study and their objectives. They accepted to participate in our study. The study was conducted according to the World Medical Association Declaration of Helsinki and was approved by the Ethics Committee of the Universitat Politècnica de València.

### Statistical tests

The normality of the quantitative data was verified using the Shapiro–Wilk test (Patrício et al. 2017). The data did not come from a normal distribution. Therefore, nonparametric tests were used (Mann–Whitney *U* test, Kruskal–Wallis test, and Spearman's correlation). The data provided by these tests is presented using the format (statistic *U*, approximation to the normal *Z*, *p* value, *r* effect size). A statistically significant difference is verified for *p* less than 0.05 and is indicated by adding the symbol '**'.

## Results

### Performance measures

**Fig. 5:**
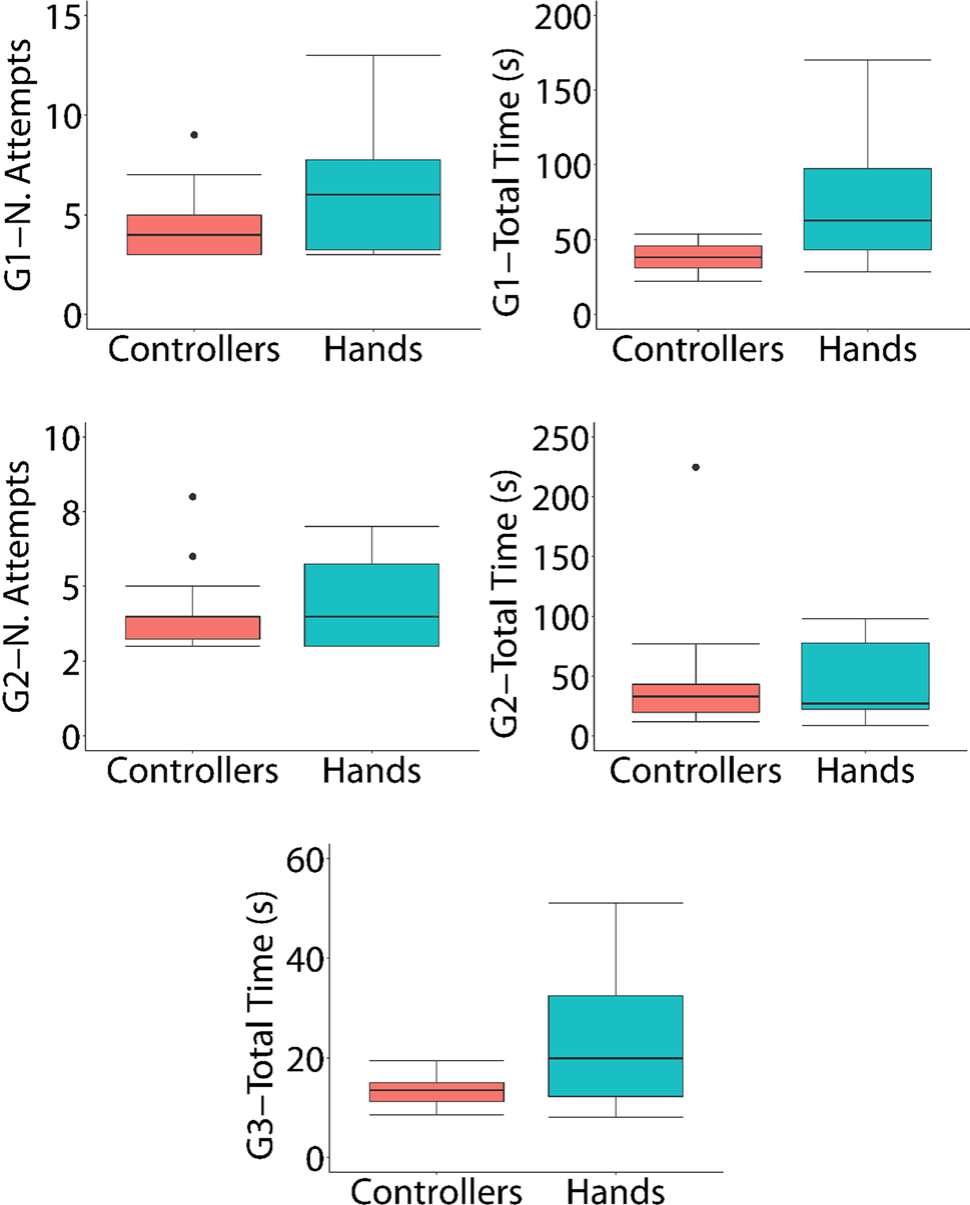
Box plots for the three games and the *Number of Attempts* and *Total Time* variables

To determine how the type of interaction affects the performance outcomes, we compared the performance outcomes between the two conditions (Controllers vs. Hands) for their first contact with the application (between-subject analysis). Figure 5 shows the box plots for the three games and the *Number of Attempts* and *Total Time* variables. In the first game, for the *Number of Attempts variable*, the Mann–Whitney *U* test was applied (*U* = 64.5, *Z* = −1.572, *p* = 0.121, *r* = 0.297). This result indicates that there are no statistically significant differences between the two conditions. If the outlier (shown in Fig. 5) is eliminated from Group A (controllers), the result still indicates that there are no significant differences between the two conditions. This outlier does not correspond to any participant with motor problems. For the *Total Time* variable, the Mann–Whitney *U* test was also applied (*U* = 46, *Z* = −2.389, *p* = 0.016**, *r* = 0.452). This result indicates that there are significant differences in favor of the condition that used the controllers, which required less time.

In the second game, for the *Number of Attempts* variable, the Mann–Whitney *U* test was applied (*U* = 95, *Z* = −0.144, *p* = 0.905, *r* = 0.027). If the two outliers (shown in Fig. 5) are eliminated from Group A (controllers), the result still indicates that there are no significant differences between the two groups. These two outliers correspond to two participants with motor problems (one with osteoarthritis and one with essential tremors). For the *Total Time* variable, the Mann–Whitney *U* test was applied (*U* = 92, *Z* = −0.276, *p* = 0.804, *r* = 0.052). If the outlier of Group A (Controllers) is eliminated, the result still indicates that there are no significant differences between the two conditions. This participant had essential tremor problems. These results indicate that for the second game there are no significant differences between the two conditions for the two variables (*Number of Attempts* and *Total Time*).

In the third game, there is no *Number of Attempts*. For the *Total Time* variable, the Mann–Whitney *U* test was applied (*U* = 61, *Z* = −1.700, *p* = 0.094, *r* = 0.321). This result indicates that there are no significant differences between the two conditions.

From these results, it can be deduced that the participants required more time when using their hands in the first game. However, after habituation in the first game, the following two games were played without significant differences in terms of attempts and time spent. With respect to the participants with motor problems, it can be concluded that only the performance in the second game differed from the rest of the participants. Some of them required more attempts and time to complete this second game.

### Gender and age

**Fig. 6:**
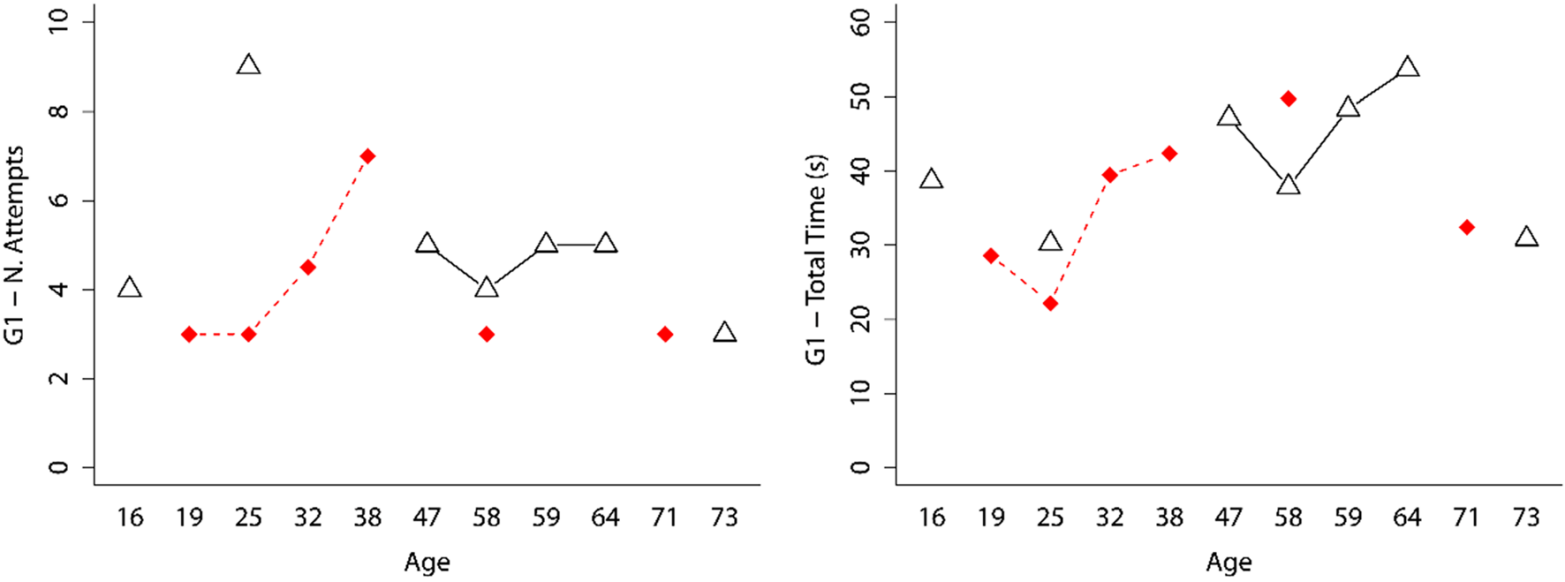
Interaction graphs (age and gender) for the first game, using controllers and for the *Number of Attempts* and *Total Time* variables. Men are indicated by a red rhombus and women by a white triangle

**Fig. 7:**
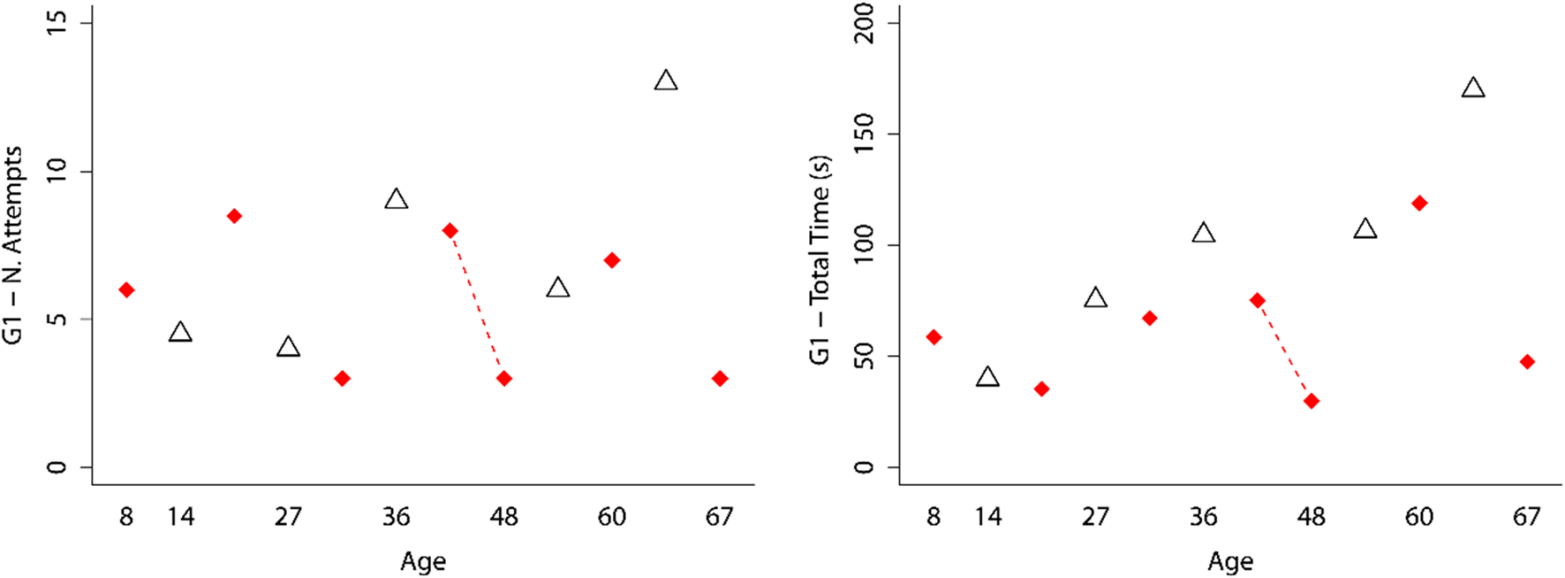
Interaction graphs (age and gender) for the first game, using hands and for the *Number of Attempts* and *Total Time* variables. Men are indicated by a red rhombus and women by a white triangle

Figures 6 and 7 show interaction graphs (age and gender) for the first game, using controllers or hands, respectively, for the *Number of Attempts* and *Total Time* variables.

The Mann–Whitney *U* test was applied to determine if gender (group of men vs. group of women) affected the *Number of Attempts* and *Total Time* variables, the first time that they used the games and taking into account whether the participants used controllers or hands. The results are shown in Tables 1 and 2, respectively. As can be observed in these tables, no significant statistical differences were found for any of the variables analyzed. Therefore, it can be concluded that the performance outcomes were independent of the gender of the participants.

**Table 1:** Mann–Whitney *U* test for the *Number of Attempts* and *Total Time* variables of the group that used the controllers for their first use for the three games and taking into account gender (men vs. women)

Game-variable	*U*	*Z*	*p*	*r*
G1-N. Attempts	14.5	− 1.328	0.207	0.355
G1-Total Time (s)	19.0	− 0.703	0.535	0.188
G2-N. Attempts	16.5	− 1.106	0.300	0.295
G2-Total Time (s)	14.0	− 1.342	0.209	0.359
G3-Total Time (s)	21.0	− 0.447	0.710	0.120

**Table 2:** Mann–Whitney U test for the *Number of Attempts* and *Total Time* variables of the group that used their hands in their first use for the three games and taking into account gender (men vs. women)

Game-variable	*U*	*Z*	*p*	*r*
G1-N. Attempts	20.5	− 0.462	0.692	0.124
G1-Total Time (s)	14.0	− 1.291	0.228	0.345
G2-N. Attempts	26.0	0.270 (Note:align the numbers in this column)	0.840	0.072
G2-Total Time (s)	27.0	0.387	0.755	0.104
G3-Total Time (s)	26.0	0.258	0.852	0.069

To determine whether age influences the *Number of Attempts* and *Total Time* variables, the Kruskal Wallis test was applied to the groups that used controllers or hands for their first use. The results are shown in Table 3. In all of these analyses, no statistically significant differences were found. Therefore, it can be concluded that the performance outcomes were independent of the participants’ age.

**Table 3:** Kruskal Wallis test for the *Number of Attempts* and *Total Time* variables for the groups that used controllers or hands for their first use

Game-interaction	Variable	df	*H*	*p*
G1-controllers	N. Attempts	10	8.391 (Note: align the numbers in this column)	0.591
G1-Controllers	Total Time	10	11.371	0.329
G2-controllers	N. Attempts	10	11.989	0.286
G2-controllers	Total Time	10	11.000	0.358
G3-controllers	Total Time	10	4.029	0.946
G1-hands	N. Attempts	11	11.349	0.415
G1-hands	Total Time	11	12.286	0.342
G2-hands	N. Attempts	11	9.875	0.542
G2-hands	Total Time	11	8.857	0.635
G3-hands	Total Time	11	10.657	0.472

### Subjective measures

**Fig. 8:**
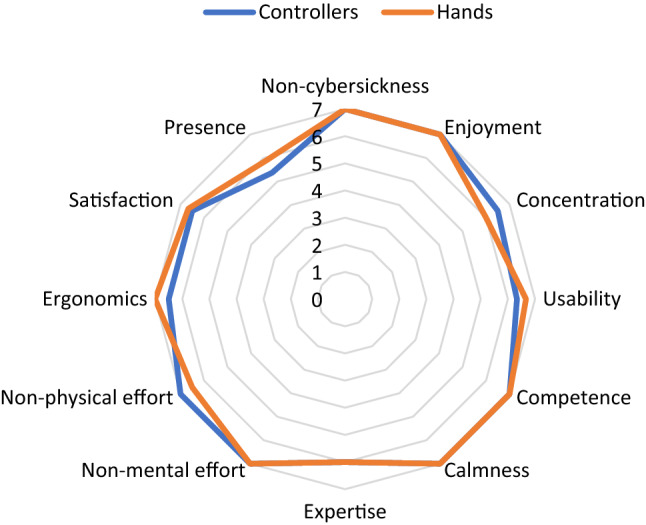
Radial graph showing the medians of the subjective variables

Figure 8 shows a radial graph with the means of the subjective variables from the questionnaire that was filled out by the participants after the first use of the games using controllers or hands. As can be observed, practically all of the medians are > 6 (values between 1 and 7). There are only two medians that are > 5 and < 6. Table 4 shows the results after applying the Mann–Whitney *U* test to the different subjective variables and comparing the two types of interaction. The results indicate that there are no statistically significant differences for any of the subjective variables. These analyses indicate that the games and the two types of interaction have been widely accepted by users.

**Table 4:** Mann–Whitney *U* test for subjective variables and taking into account the type of interaction (controllers vs. hands)

Subjective variables	*U*	*Z*	*p*	*r*
Non-cybersickness	73.0 (Note: align the numbers in this column)	− 1.512 (Note: align the numbers in this column)	0.138	0.286
Enjoyment	90.0	− 0.431	0.686	0.081
Concentration	110.5	0.591	0.571	0.112
Usability	74.5	− 1.101	0.281	0.208
Competence	111.0	0.708	0.496	0.134
Calmness	112.0	1.441	0.165	0.272
Expertise	98.0	0.000	1.000	0.000
Non-mental effort	119.0	1.454	0.156	0.275
Non-physical effort	123.0	1.350	0.186	0.255
Ergonomics	82.0	− 0.804	0.436	0.152
Satisfaction	93.0	− 0.243	0.827	0.046
Presence	77.5	− 0.947	0.355	0.179

The score for the different questions of the participants with motor problems was not significantly different from the rest of the participants.

### Correlations

Tables 5 and 6 show the significant Spearman correlations obtained between the performance and the subjective measures for the first use using controllers and hands, respectively. The results of Tables 5 and 6 show that no significant correlations were obtained between the performance variables and the subjective variables for participants who used the controllers for their first use. In contrast, there are significant correlations between the performance variables and the subjective variables for users who used their hands for their first use. From the results, it can be observed that, the greater the number of attempts and the greater the time required, the less usability and the less perceived competence.

**Table 5:** Spearman correlation

Variables	2	3	4	5	6	7
1. Non-cybersickness	0.58**	0.43	0.36	− 0.51*	0.0 (Note: align the numbers in this column)	0.61** (Note: align the numbers in this column)
2. Enjoyment		0.72**	0.36	− 0.34	0.22	0.71**
3. Concentration			0.29	− 0.36	0.29	0.48
4. Calmness				− 0.32	0.58*	0.15
5. Expertise					− 0.45	− 0.08
6. Non-physical effort						− 0.02
7. Satisfaction						1.00

Significant correlations between the subjective and performance measures for the first use using controllers. The meaning of the numbers in the rows is associated with the numbers in the columns. Only variables that have at least a significant correlation are shown.

*Indicates marginal correlation, *p* < 0.067

**Indicates *p* < 0.05

**Table 6:** Spearman correlation

Variables	2	3	4	5	6	7	8
1. Sum of N. of Attempts	0.78**	− 0.57**	− 0.06 (Note: align the numbers in this column)	− 0.53* (Note: align the numbers in this column)	− 0.72* (Note: align the numbers in this column)	− 0.19 (Note: align the numbers in this column)	− 0.47 (Note: align the numbers in this column)
2. Sum of Total Time		− 0.61**	− 0.19	− 0.54**	− 0.52*	− 0.30	− 0.41
3. Non-cybersickness			0.29	0.57*	0.40	0.16	0.15
4. Concentration				0.27	0.34	0.51*	0.54**
5. Usability					0.59**	0.57**	0.43
6. Competence						0.50	0.51*
7. Satisfaction							0.66**
8. Presence							1.00

Significant correlations between the performance and subjective measures for the first use using hands. The meaning of the numbers in the rows is associated with the numbers in the columns. The Sum of N. of Attempts variable is the sum of the attempts from all three games, and the Sum of Total Time variable is the sum of the time spent in the three games. Only variables that have at least a significant correlation are shown.

*Indicates marginal correlation, *p* < 0.067

**Indicates *p* < 0.05

Table 7 shows whether the user habitually uses electronic devices to play, their familiarity with virtual reality or headsets, and the correlations with the subjective and performance variables for the first use with the controllers. When using hands for the first time, only three significant correlations were found between: familiarity with devices for playing games and enjoyment (*ρ* = −0.53, *p* = 0.049); familiarity with VR and usability (*ρ* = 0.81, *p* < 0.001); and (marginal correlation) familiarity with headsets and comfort when wearing the headset (*ρ* = 0.52, *p* = 0.056). These results highlight the fact that familiarity with gaming devices influences the user to need less time to complete the games, requires less concentration, and provides a greater sense of expertise. Familiarity with VR applications or headsets produces less enjoyment. Our explanation for this last result is that the novelty effect does not arise in users with this familiarity.

**Table 7:** Spearman correlation

Variable	F. gaming devices	F. RV	F. HMDs
Familiarity RV	0.59** (Note: align the numbers in this column)	(Note: align the numbers in this column)	(Note: align the numbers in this column)
Familiarity HMDs	0.35	0.84**	
Total Time	− 0.59**	− 0.30	− 0.13
Non-cybersickness	− 0.42	− 0.72**	− 0.59**
Enjoyment	− 0.38	− 0.71**	− 0.60**
Concentration	− 0.56**	− 0.54**	− 0.41
Calmness	− 0.26	− 0.47	− 0.51*
Expertise	0.59**	0.59**	0.34
Non-physical effort	− 0.49	− 0.57**	− 0.53*
Satisfaction	− 0.37	0.54**	− 0.41

Significant correlations between the variables of familiarity with the technology and the performance and subjective variables for the first use using the controllers

**Indicates *p* < 0.05

*Indicates marginal correlation, *p* < 0.067

### Preferences

This section focuses on users’ preferences and includes some of their comments. The questions were: 1) Which type of interaction do you prefer (controllers or hands?); 2) Why? A total of 78.5% of the users (22 out of 28) showed their preference for interaction using their hands. Some of their comments were the following: “It is easier to move your own hand than a device in your hand”; "It is more natural"; "It is more real"; "It is more intuitive"; "You don't have to carry anything, and you don't have to carry weight." In contrast, the most used argument in favor of the preference for the use of controllers was the following: “It is more precise”.

All four users with motor problems selected their hands as the preferred type for interaction and argued its resemblance to reality, which is more natural and realistic.

## Discussion

The three VR serious games are different from previous systems in terms of hardware (Wen et al. 2014; Cho et al. 2016; Ballester et al. 2017; Wang et al. 2017; Aşkın et al. 2018; Oña et al. 2018; Colombo et al. 2019; Dias et al. 2019; Reggente et al. 2020; Takeo et al. 2021). The study is also different from previous works (Dias et al. 2019) since it compares gestural interaction and the use of controllers. A study involving 28 participants was carried out, which suggest that these three games may be satisfactory to perform motor rehabilitation exercises. All of the participants managed to complete the exercises proposed in the three games. No significant differences were found in the number of attempts to complete the games using the two types of interaction. If the time used to complete the games is considered, no significant differences were found to complete the second and third games using the two types of interaction. However, significant differences were found for the first game in favor of the group that used the controllers, which required less time. The results partially corroborate hypothesis H2 “There will be no significant differences in the performance of the participants when using controllers or hands”. Our argument for this result is that, although at first glance it might seem contradictory, as a general rule, users require more adaptation time to use their hands. However, after the habituation acquired using the first game, the next two games were played without significant differences in terms of attempts and time spent. To solve this issue, a more complete adaptation phase should be carried out in future experiments. This is also supported by the fact that several users commented that they would have preferred a longer adaptation phase.

Considering factors such as gender or age, and analyzing the *Number of Attempts* and *Total Time* variables, the analyses indicate that the performance outcomes were independent of the gender and age of the participants. For this reason, hypothesis H3 was corroborated "There will be no significant differences in the performance of the participants during the study based on their gender".

The questionnaires filled out after the exercises indicate the users' satisfaction with the Oculus Quest and the games, corroborating hypothesis H1 "Users will rate the games positively". There were no statistically significant differences in the participants' assessment for subjective variables when using controllers or hands. This indicates that the participants perceived both types of interaction as being suitable for motor rehabilitation. However, when the participants were asked about their preference, they mostly answered in favor of the interaction using their hands, especially the four participants with motor problems, all of whom preferred hand interaction. Moreover, several users expressed their conviction that, after an initial adaptation process, they would be more comfortable with their hands rather than with controllers. Therefore, hypothesis H4 “Participants will express their preference for the use of their hands” is also corroborated.

With regard to the preference and comments of the participants, they all expressed some positive reactions toward the environments chosen for carrying out the exercises (gym, field, house). They also expressed their interest in exploring this technology for different uses, which also corroborates hypothesis H1 "Users will rate the games positively". Furthermore, their comments include suggestions for the use of this technology in medicine, military, leisure, and education, etc. Only one participant was skeptical that this type of technology could replace tasks that are currently performed in a traditional way.

We would like to add that the version of hand tracking used had some limitations. Natural closure of an empty hand (in the form of a fist) was not identified because when the thumb was hidden under the other fingers, the application was unable to detect it as being closed. Similarly, if the thumb was left outside the fist, the application also did not detect it as being closed. Therefore, as was mentioned in the materials and methods section, for the third game, the participants were given instructions to perform the appropriate gesture so that the hand tracking was identified. With these instructions, in general, the participants had no problem performing the exercise. The gestures for the first and the second games do not differ when using hands with or without controllers. Therefore, depending on the gestures that the hand tracking is able to recognize, the gestures that can be recognized should be used and those gestures should be as similar as possible to the ones used in real life.

Four participants with motor problems participated in our study. For the first game, taking into account the *Number of Attempts* variable, the outlier shown in the box plot of Fig. 5 does not correspond to any participant with motor problems. For the second game, the two outliers shown in the box plot of Fig. 5 correspond to two participants with motor problems (one with osteoarthritis and one with essential tremors). For the second game, taking into account the *Total Time* variable, the outlier shown in the box plot of Fig. 5 corresponds to the same participant with essential tremors in the case of the *Number of Attempts* variable. These results indicate that only two of the participants with motor problems found significant differences in one of the three games (the second game) and for the *Number of Attempts* variable. Significant differences were also found for another user in the same game (the second game) and for the *Total Time* variable. However, all of the participants with motor problems were able to finish the exercises. As discussed above, all of them preferred using their hands to using the controllers. Our proposal has been very well received by these participants and the therapists involved in our research. These participants expressed their motivation to use our relaxed and fun environments. These conclusions are in line with previous works (Dias et al. 2019). Moreover, the tests with these four participants and previous studies (Ballester et al. 2017; Wang et al. 2017; Aşkın et al. 2018) suggest that our proposal can be useful for motor rehabilitation. However, a formal study with subjects with motor problems would be necessary to corroborate these preliminary results. In that future study, the advantages offered by our application could be determined and the characteristics of the patients for whom our application could be used could be identified. That study, could also identify the changes to be included for patients with different characteristics.

## Conclusion

This paper presents the design, development, and validation of the first VR application for motor rehabilitation using Oculus Quest and hand interaction. Oculus Quest is a standalone device that allows freedom of movement without having to be physically connected to any other device. This makes it easier for patients to use at home or in rehabilitation centers and other facilities. A study involving twenty-eight participants (four of whom had motor problems) was carried out to compare two types of interaction (controllers vs. hands). From the results, it can be concluded that both types of interaction allowed the exercises to be completed, without differences in the number of attempts. Furthermore, the performance outcomes using the application and the two types of interaction proved to be independent of the gender and age of the participants. However, the participants mostly expressed their preference for the use of their hands, especially all of the participants with motor problems. These results support our argument that the development of systems for motor rehabilitation that allow patients to interact in the most natural way possible would be especially accepted by patients. Moreover, interaction with hand gestures in conjunction with standalone headsets could help patients complete exercise therapy at home.

A future work consists of the inclusion of the two remaining exercises proposed by Enjalbert and the addition of gamification elements in all of the exercises (e.g., score). Another future work is to provide feedback in the exercises to guide the user in the event that they are not doing the exercises correctly. For example, in the exercise of eating the apples, the user's head pose can be monitored to provide feedback in case of head tilt. In addition, more stimuli could be incorporated to the games in order to improve immersion (e.g., ambient and effect sounds, vibration of the controllers, or any other interactive response).

## Data Availability

The datasets generated during and/or analyzed during the current study are available from the corresponding author on reasonable request.
